# A qualitative process evaluation of universal free school meal provision in two London secondary schools

**DOI:** 10.1186/s12889-023-15082-3

**Published:** 2023-02-09

**Authors:** Patricia E. Jessiman, Victoria R. Carlisle, Katie Breheny, Rona Campbell, Russell Jago, Marcus Robinson, Steve Strong, Judi Kidger

**Affiliations:** 1 Department of Population Health Sciences, Bristol Medical School, University of Bristol, Bristol, UK; 2 Centre for Exercise, Nutrition & Health Sciences, School for Policy Studies, University of Bristol, Bristol, UK; 3 Applied Research Collaboration West (NIHR ARC West), The National Institute for Health Research, University Hospitals Bristol and Weston NHS Foundation Trust, BS1 2NT Bristol, UK; 4 London Borough of Hammersmith and Fulham, Hammersmith , UK; 5 Public co-applicant, PHIRST Insight, Hampshire, UK

**Keywords:** Food insecurity, Nutrition, School, School Meals, Qualitative, Process evaluation, Adolescence

## Abstract

**Background:**

In the UK, one in five households with children experienced food insecurity in 2022, defined as a household-level economic and social condition of limited or uncertain access to adequate food. Free school meals are a public health intervention aimed at reducing food insecurity amongst children. The provision of universal free school meals (UFSM) to secondary school-aged children is a novel and untested intervention in the UK. This study is a process evaluation of a pilot of UFSM in two secondary schools in England. The aim was to understand the feasibility, acceptability, cost implications and lessons for the implementation of UFSM.

**Methods:**

20 parents, 28 students and 8 school staff from two intervention schools participated in online qualitative interviews, as well as 4 staff from non-intervention schools. The Framework Method of thematic analysis was applied. These data were supplemented with student-led observations of school meal times, and school lunch uptake-data and cost information provided by the local authority delivering the pilot.

**Results:**

UFSM in secondary schools is a feasible and acceptable intervention, with coherent goals of increased access to a healthy meal, reduced food insecurity and better nutrition. All participants perceived these goals were met. Acceptability was further enhanced by the perception that UFSM were supporting a greater proportion of low-income families than the national, targeted Free School Meal scheme, as well as being easier to implement. Potential barriers to implementation include limited school kitchen and dining infrastructure, meal quality and choice, and increased queuing times. Participants’ concerns that UFSM may benefit middle- and high- income families not in need were not as prevalent as the perception that UFSM was an effective way to support all families with secondary-aged children experiencing food insecurity.

**Conclusion:**

This small-scale pilot study suggests that UFSM in secondary schools is feasible and acceptable, but more evidence is required from larger studies on the impact on long-term health, psychosocial and educational outcomes. Future, larger studies should also include detailed economic evaluations so this approach can be compared with other possible interventions.

**Supplementary information:**

The online version contains supplementary material available at 10.1186/s12889-023-15082-3.

## Background

The United Nations defines household food security as a situation in which all those in the household have physical, social and economic access to sufficient, safe and nutritious food at all times that meets dietary needs and food preferences for an active and healthy life [1]. The Trussell Trust, a third sector organisation supporting a network of more than 1200 foodbanks across the UK, define household food *insecurity* as ‘a household-level economic and social condition of limited or uncertain access to adequate food’ [2]. Food insecurity and child hunger has long been a problem in the UK, and has been a growing political and social concern in the decade running up to the outbreak of the COVID-19 pandemic in early 2020 [3].

Food insecurity in the UK has worsened during the COVID19 pandemic. One UK study suggests that in April 2020, just after the first lockdown and school closure, only around half of children eligible for free school meals actually received them [4]. The Food Foundation reported that one in five households with children experienced food insecurity in the six months leading to April 2022 [5], while the UK Food Standards Agency found a similar proportion of people in the UK (22%) report skipping or reducing meal size because they did not have enough money to buy food [6].

Experiencing food insecurity in childhood has health and social consequences. Food insecurity is associated with poorer diet quality in children [7]. In adolescence, nutritional intake influences the onset and timing of puberty, which in turn influences height, muscle and fat mass, and the risk of non-communicable diseases in adulthood [8]. In a rapid review of child food insecurity, Aceves-Martins et al. found 74 studies that assessed the effects of food insecurity on children and found evidence of impact on physical health status (including chronic conditions), social well-being, mental and emotional health (e.g. externalising and internalising behaviours, aggression, hyperactivity, impaired social skills) and academic outcomes [9]. There was mixed evidence of the impact on weight status; some studies report food insecurity is associated with obesity while others find no association, or one that is mediated by sex, age, psychosocial or parental characteristics.

Free school meals (FSM) are a public health intervention aimed at reducing food insecurity and increasing nutritional status amongst children. Historically in the UK, FSM have been means-tested, with universal provision of free meals a relatively new and developing policy targeted towards younger children. In 2015 Scotland moved from a means tested system for FSM to a universal system for children in Primary 1–3 (when most students are aged 5–8 years) and more recently the Scottish Government extended universal provision up to Primary 5 [10]. The Welsh Government recently announced plans to offer FSM to every primary school pupil from September 2022 [11]. In Northern Ireland, FSM remains a mean-tested benefit for all ages. In England, universal FSM are currently provided for all children in reception up to and including Year Two (4–7 years). Beyond that age, FSM are a targeted benefit. Children of all ages in full time education are eligible for free school meals if their parents/carers are in receipt of state benefits such as Child Tax Credit or Universal Credit although the eligibility threshold is high (gross income must be less than £16,190 per annum for those in receipt of child tax credit and below £7,400 for those in receipt of Universal Credit) [12]. In England, over one in five (22.5%) of pupils are eligible for FSM [13].

There is some evidence for the beneficial impact of universal free school meal provision (UFSM) on student health and educational outcomes. In one review of the effectiveness of interventions to mitigate the effects of food insecurity in high income countries, six of fifteen included studies were of school-based food provision, most conducted in the USA. Interventions were effective in improving but not eradicating food insecurity and its effects on health and academic outcomes [9]. A recent systematic review of UFSM, in which 47 studies were included, found positive associations with increased meal participation rates, improved nutrition and academic performance (where UFSM included lunch rather than breakfast), and improved food security [14]. A cross-sectional study of 311 students aged 4–11 years in England found that FSM were more likely to contribute to a healthy diet than packed lunches [15], although a similar study of 4–7 year old pupils in England suggests the inclusion of cakes and sweet puddings as part of the lunch offer may limit the dietary benefit [16].

This paper is concerned with the implementation of UFSM provision in two secondary schools in a London Borough (local government administrative area). As universal FSM has been limited to younger children, the provision of universal free school meals to secondary school-aged children (aged 11–16) is a novel and untested intervention in the UK. Given the uniqueness of the intervention and the rising concerns about child food insecurity, the local authority applied to the UK National Institute for Health Research (NIHR) Public Health Interventions Research Studies Teams (PHIRST) initiative for evaluation support [17]. A protocol for the mixed-method quasi-experimental impact evaluation with embedded process evaluation is available [18], and findings of the impact study forthcoming.

There is little literature on the feasibility and implementation of UFSM, particularly for older students. A process evaluation of the introduction of UFSM to younger primary pupils in Scotland found that while the policy was implemented successfully, schools and local authorities faced continuing challenges with recruiting and training staff to prepare meals and supervise children effectively, adapting and improving kitchen and dining areas to cope with increased uptake, and engaging parents with the policy, including feedback on quality and menu options. Some senior school managers did not agree that UFSM was the correct policy to support families most in need, which may also have hampered implementation [19]. Understanding the feasibility of this UFSM pilot may help identify whether similar (or additional) barriers arise in secondary schools which may hinder the acceptability and implementation of any wider roll out [20, 21]. It is also important to determine whether UFSM has the potential to be a cost-effective public health intervention. Though the small scale of the current pilot does not allow for a full economic analysis, determining the costs and the acceptability of these costs may influence the adoption of the policy more widely [22].

This paper reports the results of a process evaluation of the UFSM pilot designed to answer the following research questions:

R1. Is UFSM feasible and acceptable in a UK secondary school context?

R2. What are the enablers and barriers to the effective implementation of UFSM in secondary schools?

R3. What are the cost implications of implementing UFSM in secondary schools as a means of addressing student hunger?

## Methods

### Pilot schools

The local authority agreed the budget for the pilot in Summer 2019 and delivery commenced in January 2020. The two schools involved were relatively small secondary schools in inner London; School 1 had approximately 400 students at the start of the intervention and School 2, which provides education for students with special educational needs and disabilities, around 100 students. The schools were selected by the local authority prior to the start of the study based on their high level of need (both schools have FSM eligibility above the national average, see Table 1) and capacity to deliver the pilot. Characteristics of the two intervention schools at the start of the intervention are shown in Table 1 below.

**Table 1:** Intervention school characteristics

	School 1	School 2
School type	Mainstream	Special School
No of Pupils	414	105
Eligibility for national Free School Meal scheme (%)	33.9	46.6
% Boys	77.8*	69.7
% non-white	77.1	56.4

*School 1 was, until recently, an all-boys school.

### Study design

We conducted a qualitative process evaluation collecting data through interviews with students, their parent or carers (hereafter ‘parents’), and school staff from two intervention schools delivering UFSM. These data were supplemented by student observations of school lunches during the UFSM pilot, and interviews with school staff from non-intervention schools in the same borough in which UFSM was being delivered. Data were collected between June 2021 and January 2022. As the study was designed during the COVID-19 pandemic, data were collected remotely.

Staff from Children’s Services, Public Health, and Adult Services involved in commissioning UFSM and other aspects of school meal delivery were involved in the development of the study protocol and design of data collection tools. The study team also undertook public involvement work with parents and students to support the development of data collection methods and tools that were relevant, inclusive and accessible. This included consulting with a small group of parents living in the local authority area about the participant information sheets, topic guide and recruitment strategy before data collection began, and conducting two online workshops with school students to co-design the tools to be used in the peer observation element of the study.

Prior to the interviews and observations, participants were sent a participant information sheet (PIS) detailing the aims of the study, and what taking part would involve including the use of data and confidentiality. Ethical approval for the study was awarded by the University of Bristol’s School for Policy Studies Research Ethics Committee in March 2021 (ref. SPSREC/20–21/151). All participants provided written informed consent; student consent forms were co-signed by parents.

### Parent interviews

The study aimed to recruit parents across the two intervention schools to participate in an in-depth qualitative interview. Parents were recruited by the school sending out detailed participant information sheets and a pre-interview questionnaire and invited to return the questionnaire and contact details directly to the research team if they wanted to participate. The questionnaire included questions about eligibility for free school meals, food affordability, hunger and meal-skipping for both adult and children in the household using the USDA Household Food Security Survey Module (HFSSM) scoring system [23, 24]. In this measure, ‘low food security’ means that the household have to reduce the quality, variety, and desirability of their diets, and ‘very low food security’ means that household members sometimes disrupt eating patterns or reduce food intake because they lack money or other resources for food. A purposive sampling strategy was adopted using: (a) eligibility for FSM under the national scheme; (b) household food security; (c) ethnicity of child.

Interviews were undertaken either online or by telephone. Parents were offered the use of a translator if required (it was not). A topic guide was developed that explored the following areas: background information about the family, experience of food insecurity, school lunch arrangements before and after the introduction of UFSM, uptake (or not) of UFSM, perceptions of UFSM and its impact(s) including unintended consequences, and differential impact of UFSM compared to the national FSM scheme. Interviews lasted 40 min on average (range 28-68 min) and were recorded verbatim on an encrypted digital recorder. Parents were offered a £30 voucher as a thank you for their time and input.

### School staff interviews

We aimed to recruit up to six staff from each of the intervention schools. These included members of the senior leadership team; teaching staff (including those with pastoral support responsibilities and/or lunchtime supervision duties as part of their role), and catering staff. We also aimed to recruit up to eight senior leadership staff from non-intervention schools in the same local authority. The topic guide for intervention school staff included questions on demographics of school students and perceptions of school lunch uptake, the national free school meal scheme, lunch arrangements pre- and post- UFSM implementation, concerns about food security amongst students, impact of UFSM, and questions about the implementation, cost-effectiveness and feasibility of UFSM. The topic guide for non-intervention school staff included questions on the feasibility of implementing UFSM in their school and its perceived impact on the school staff, students and families. Questions were included on the acceptability of UFSM, and any preferred alternative approaches to addressing food insecurity which they would consider if funding were available. Again, these took place either online or by telephone and were securely recorded. Catering staff were offered a £30 voucher as they took part in their own time. Costs for school leadership and teaching staff time were reimbursed to the school at £100 per interview.

### Student observation diaries

Structured observations of mealtimes have been undertaken in other studies of universal provision, for example in an evaluation of UFSM for all children in Primary 1 to 3 (ages 5–8 years) in Scotland [19]. However, COVID-19 restrictions precluded the research team from undertaking direct observations in schools. Instead, we recruited members of the student councils in each participating school to act as co-researchers to undertake structured observations.

The researchers, with the support of school staff, met online with students from both schools to co-design a diary instrument that could be used to record lunchtime observations. Students were asked what aspects of school lunch were important to them and should be considered in the study. Their responses shaped the design of a tool that included questions on the dining hall environment, menu options, student food choices, portion size, perception of quality, serving and eating of meals, and other lunchtime activity. The tool was piloted in both schools. A sample observation diary can be seen in Appendix 1. Students were asked to use the tool to conduct lunchtime observations over five school days. The diary instrument was the same for both schools (staff and students at the SEN school did not request any amendments; however in practice students from School 2 took fewer photos and wrote fewer comments than School 1 students).

### Student interviews

In-depth qualitative interviews were undertaken with students from each school. Our intended sampling criteria included eligibility for FSM using national criteria; year group; take-up of UFSM provision; and ethnicity. However, due to disruption to school attendance as a result of the COVID-19 pandemic (including school closures, students kept home for isolation, and COVID-related absence) convenience sampling was adopted. Information about the research was shared with all students and parents in each school. Students were asked to let a member of school staff know if they would like to take part, and staff then supported the selection of students from a range of year groups and ethnicities.

The qualitative interviews took place online. Students were offered the opportunity to bring a friend to the interview for support and reduce any power imbalance with the researcher. None chose to, but in one school interviewees were paired at the request of the school to minimise disruption to the school day. In the other school, all interviews were conducted with a single respondent. The topic guide included questions on school lunch arrangements pre- and post UFSM (and reasons for uptake of school lunch or not), pre- and post-school food consumption and snacking, perceptions of UFSM including quality, choice, and lunchtime arrangements, and perceptions of impact of UFSM, including unintended consequences. Students were given a £15 voucher as a thank you for participation. The interviews were securely recorded.

### Qualitative analysis

All interview data were transcribed verbatim, and analysed using the Framework Method of thematic analysis using Nvivo software [25, 26]. Student, parent and staff datasets were analysed separately. For each set of data, one of the research team developed a draft thematic framework based on the research questions and after reading several transcripts. This draft framework informed an initial coding stage, and the framework was refined by adding subthemes that were identified in further transcripts. A short summary of each subtheme was developed to describe the data that it was designed to capture. This Framework was shared with the whole research team and then further refined until the team were confident that it encompassed all the data in the transcripts, the data within each subtheme were coherent, and there were clear distinctions between subthemes. Consistency and agreement were achieved by at least two researchers comparing the application of subthemes with a small number of transcripts.

We created matrices for each data set in which cells were populated with verbatim and summarised data from the transcripts, showing data from every respondent under each subtheme, thus providing a detailed and accessible overview of the qualitative dataset. A summary of the data under each subtheme was developed to inform the next stage of the analysis, moving up the analytical hierarchy to explore patterns and associations between themes in the data. Themes identified in students, parents and staff were compared and contrasted. Findings were also compared across the two schools, but few differences were apparent; where findings differed, we highlight these in the results section.

We present the qualitative data relating to feasibility using Sekhon *et al’s* theoretical framework of acceptability [27]. This was done because acceptability has tended to be conflated with ‘satisfaction’ in healthcare and public health intervention research; the Sekhon framework goes beyond this to include seven components: affective attitude, burden, perceived effectiveness, ethicality, intervention coherence, opportunity costs, and self-efficacy. Developed to assess both prospective and retrospective acceptability of those involved in both the delivery and receipt of an intervention, it is widely cited in health intervention studies [28]. Other aspects of feasibility are also addressed in a review of the enablers and barriers to implementation.

### School lunch uptake and cost data

The local authority provided data on the number of lunches served per month in the intervention schools from the intervention start date (January 2020) until March 2022. The latest data provided to the research team was for March 2022.

Costs of funding UFSM were also provided by the local authority for the 2020–2021 and 2021–2022 financial years. Intervention schools paid the external catering companies and were refunded by the local authority for the cost of providing UFSM. The cost of each meal reimbursed was fixed at £2.30. The cost of providing UFSM for one child for one academic year was also estimated, assuming a 195-day school year, and 100% uptake and attendance.

## Results

In total, 60 respondents took part in an interview, and 59 observation forms were returned by the student observers. The sample included 20 parents of students attending the two intervention schools. Half of this sample had previously been eligible for the national free school meal scheme, and half were experiencing low levels of food security at the time of interview. Of the 12 school staff interviewed, eight were drawn from the intervention schools and four from non-intervention schools (2 from a special school, 1 from a mainstream academy and 1 from an all-girls school)The sample included six members of the senior leadership team (e.g. head or deputy head); four teaching staff and two catering staff. Twenty-eight students were interviewed, from across Years 7–11. Information about the sample of parents, students, and school staff can be seen in Tables 2 and 3 below.

**Table 2:** Parent and Student Sample

**Parent Sample (N = 20)**		
	School 1 (n = 13)	School 2 (n = 7)
Eligible for the national FSM scheme	5	5
Low, or very low food security status	4	6
Ethnicity of child:		
Black	2	3
White	5	2
Asian	2	-
Mixed	2	2
Other	2	-
**Student sample (N = 28)**		
	School 1 (n = 18)	School 2 (n = 10)
Year 7 (aged 11–12 years)	4	1
Year 8 (12–13 years)	4	3
Year 9 (13–14 years)	4	2
Year 10 (14–15 years)	4	2
Year 11 (15–16 years)	2	2

**Table 3:** School Staff sample (N = 12)

	School 1 (n = 5)	School 2 (n = 3)	Non-intervention schools (n = 4)
Leadership team	2	1	3
Teaching staff	2	1	1
Catering staff	1	1	-

Student co-researchers completed 59 observation forms (Table 4). Some of these were completed by individuals, some by groups of students working together. The research team did not receive sufficient data from school staff to determine how many were completed collectively. Each observation therefore represents at least one student’s record of a lunchtime observation. Qualitative data from these observation reports have been merged with student interview data in the presentation of findings.

**Table 4:** Student peer observations

	School 1	School 2
No. of observations	39	20
No. of students involved	13	11

### Is UFSM feasible and acceptable in a UK secondary school context?

#### Affective attitude

In the Sekhon framework [27], *affective attitude* describes how individuals feel about taking part in the intervention. Staff, students and parents were universal in their support for UFSM, perceiving it as a positive intervention with benefits for all participants. Most parents were keen to see it continue (with some worried about the impact if UFSM was withdrawn), and many hoped that it would be extended to other schools. Many linked UFSM with high-profile news events happening at the time, including the COVID-19 pandemic and the challenge faced by many low-income families in feeding their children, the campaign launched by Manchester United and England footballer Marcus Rashford to address this, and the economic impact of Brexit. The financial saving was reported as a major benefit.

*I was paying. I don’t even know how much they would cost here. I think in primary school, two pounds per day, so it’s not a lot when you say, okay, two pounds. But then you have two children and it’s like five days a week, it does add up. And I don’t think I could do home lunch for cheaper…[]… and so, it is it is financially, it’s also very helpful.*

Y7 Parent School 2

School staff in the intervention schools enjoyed working in a school that participated in UFSM because of the perceived benefits to students, including ensuring access to a meal at lunchtime and reduced financial burden on families. Staff often reported concerns about families and students living with food insecurity, and the introduction of UFSM had reduced their worry about students experiencing hunger. Catering staff in particular gained satisfaction from providing for all students, particularly those in low-income families.

*It helps a lot of people out, doesn’t it, and if they’re getting a full meal and full pudding, they’re not going to go home starving. They do get a really good full meal and a full pudding. It’s bigger than a dinner, I would say, wouldn’t you? Portion and everything else. Yeah, it’s a big meal. They get a substantial meal, so I think that all children go home happy.*

Catering staff, School 1

Staff from non-intervention schools were also supportive of UFSM as a means of supporting the high numbers of children and families struggling with food insecurity.

*I only see good things out of it [UFSM] to be honest with you. The area we are based…[]… there is a lot of families that struggle around here…[]…my job is to try to remove barriers to learning and one of the number one things that I have to deal with on a weekly basis is usually sleep and food, the two things most of our student struggle with is sleep and food and when I speak to parents about the issues, some of them are saying to me “I am struggling with money, my son didn’t have dinner tonight or lunch or I couldn’t afford a packed lunch for him or her”.*

Non-intervention school staff

Most students interviewed were participating in UFSM. Those who did not and took a packed lunch to school did so because they did not think there was enough choice in school and would prefer a greater number of menu options, or preferred to eat home-cooked food. There was some dissatisfaction with the quality and quantity of food available in the intervention schools amongst students, though others spoke positively about these aspects. Most students reported that the introduction of UFSM had resulted in increased uptake of school meals in both schools, and many liked that because this meant more of their friends were likely to be in the canteen eating together. Some students also valued the time saved by them, or their parents not having to prepare a packed lunch. All students were supportive of the change to UFSM, primarily because of the money this saved them and their families. This was perceived to be especially useful to those students eligible for the national FSM scheme and those who were not but still struggled to afford meals.

*For the people who had free school meals before it’s not a weakness for them anymore because everybody has free school meals so it just makes them feel equal and not like different to everyone just because of what their family have or do not have.*

Year 9 student School 1

#### Coherence and perceived effectiveness

The positivity towards taking part in UFSM was in large part attributed to its *coherence* and *perceived effectiveness*. Respondents from all groups had a clear understanding of what the introduction of UFSM was intended to achieve; increased access to a healthy meal at lunchtime for all students, reduced food insecurity and better nutrition.

*Everybody benefits from it...[]…. Even if it’s just a handful of extra children, it’s a handful of extra children that aren’t hungry. That would impact the complete family, because it is stressful. When you’ve got children that are hungry, it is stressful.*

Y8 Parent School 1

School staff perceived that these intended outcomes were being met; the vast majority of students in both schools received a free school meal at lunchtime and the proportion of students bringing in a packed lunch was reduced. This is supported by lunch uptake data collected by each school (Table 5 below) showing that the number of lunches served per month almost doubled in two years from the intervention start date in School 1; and also increased in school 2.

**Table 5:** Uptake of UFSM in intervention schools

Uptake of UFSM (lunches per month)		
	School 1	School 2
Intervention start (Jan 2020)	2218	1314
September 2020 (schools re-open after COVID-19 closures)	2635	1357
September 2021	4490	1401
March 2022	4379	1707

School census data compiled by the local authority also indicates that, in both schools, the proportion of students eligible for FSM who accessed a free school meal increased. In November 2019, just before the start of the pilot, 55% of FSM students in School 1 took a school lunch and 74% of FSM students in School 2. In November 2021, this had increased to 78% and 79% respectively.

Staff perceived that students who had changed from packed or bought-offsite lunches to UFSM were more likely to be consuming healthier and more nutritious food. Staff also reported the increased uptake of school lunch resulted in more students eating together, often with school staff, bringing an opportunity to gain social skills and develop healthy eating habits.

*I think the pupils are socialising better at lunchtime as well. Particularly in the lunch hall it’s a calmer environment. We’ve been able to really embed a few other life skills around eating, hygiene, being able to actually access food with a knife and fork and being able to eat correctly. Role-modelling that from staff and those things have been really important…[]… they’re gonna have to start making their own food choices soon so you know, the sooner we can get them used to that this is right way to eat, that there’s some portion control as well, you know I think that’ll be beneficial longer term.*

School 2 Staff

Students and teaching staff also perceived that student behaviour and engagement had improved in the afternoons, attributing this to increased uptake of meals and fewer students going hungry or eating low-quality packed lunches.

#### Burden

Implementing UFSM brought some *burden* for participants, school staff and students in particular (parents were unlikely to report any burdens). Some students in both the interviews and observations reported burden in terms of increased queuing times and the canteen running out of some meal options before they had chance to eat.

*Our year, are the last year to get lunch. Sometimes there is no main option because everything’s finished and sometimes you cannot find cake or custard that you wanted. …[]… I was last for a bit and then I got a sandwich which was in a box. But yeah, they ran out. I’m not coming in last but the times I have been last I have noticed that, definitely.*

Year 8 student School 1.

School 2, in anticipation of increased uptake as a result of UFSM, required an upgrade to the kitchen equipment and electrical supply.

*We’d anticipated about 10 to 20 percent increase in lunches so we had to get a second fridge freezer, the dishwasher was breaking all the time, so we realised that we were damaging staff backs. The local authority came in and worked with us on that …[]… all those little things, the technical side of things had to be done before we kind of went live.*

School 2 staff.

Catering staff reported that workload increased with the implementation of UFSM, due to increased uptake and also, in one school, a change to the FSM offer to include two courses (main meal and dessert) instead of main meal and drink (drinks other than water were withdrawn from the free meal offer because of concerns about the health and climate implications of bottled drinks). This required more resource to prepare dessert for all students. Caterers increased numbers of staff to cope.

*My boss has taken on another member of staff because we’re busier. But yes, no it all seems to have run quite smoothly really. Now we’re fully staffed, now we’ve got the extra staff, it’s okay on the preparation of getting everything done.*

Catering staff School 2

Other preparatory work for school staff included informing parents about the introduction of UFSM, usually by letter or email, and encouraging parents to participate in UFSM. Participating schools needed to ensure that eligible families still applied to receive FSM, in order for the school to reclaim this money and also because the proportion of FSM-eligible students on roll has influence over other funding schools receive. Schools requested that all families completed a ‘Healthy Meals Allowance’ form, which also captured FSM eligibility information. While some staff reported that this initial communication work was resource-intensive it was not seen as barrier to the feasibility of UFSM. Once up and running, school staff reported that the ongoing management of lunches was easier for UFSM than for the national means-tested FSM scheme. The burden of dealing with students without lunch money, students borrowing from and/or bullying each other for food and money, had significantly lessened.

*It’s really great when it’s there for all of them because they forget to bring their cards into school…[]…that just being there irradicates all of the nonsense of scrabbling around. I used to lend out so many pounds. I used to come in with my pocket full of one pound coins because you’d have hungry children at lunchtime being like “Oh I forgot! I forgot to get a pound from my mum this morning,” so I’d be like giving it out and checking it in, so you know, it does save time.*

School 1 staff

#### Ethicality and opportunity cost

UFSM also appeared to correspond with the values and beliefs of most respondents (‘*ethicality’).* Students and their parents often referred to the equity of UFSM, with all students having access to the same lunch offer, at the same (no) cost. This was perceived to be especially useful to those students eligible for the national FSM scheme and those who were not but still struggled to afford meals.

*For this, for everybody having free school dinner I think it is good. Firstly, it is balanced for everybody, everybody is equal and secondly you don’t have to struggle.*

Year 7 Student School 2

*I think it’s a really nice equaliser really and it’s nice that they’re all getting it.*

Y8 Parent School 1

Some staff also reported satisfaction that the funding for UFSM had come from business rather than general taxation.

*I think the most impressive thing was the way that the local authority gained the funding to do this and that was by slapping taxes and tariffs on big building projects within the Borough.*

Staff School 2

Some parents and school staff (including in non-intervention schools) had reservations about the universality of the scheme, incurring financial benefits for mid-to-high income families who may not need them. This was perceived as less of a ‘risk’ in the intervention schools where a high proportion of children were believed to come from low-income families, many of whom would struggle to make ends meet even if they did not meet the eligibility threshold for the national FSM scheme. Nevertheless, concerns about ‘undeserving’ families benefiting and the potential waste of finite financial resources represented a challenge to the ethics and values of some participants (an *opportunity cost* in the Sekhon et al. acceptability framework [27]).

Other potential costs identified by school staff were concerns about the impact on catering companies if UFSM was implemented more widely. These centred around the potential impact on the profit margins of providers and knock-on impact of the quality of food if the funding provided to caterers for UFSM was insufficient. Catering staff also reported that continuing to sell snack food and drinks enabled their profit margins to be maintained. One respondent from a non-intervention school had introduced compulsory dining, with students restricted to the school premises at lunchtimes and barred from bring in packed lunches. All families paid for lunch unless they were eligible for FSM. Compulsory dining is unusual in UK secondary schools and was introduced to provide the school caterer with increased (and fixed) demand in an effort to increase the quality of meals, and to improve nutrition.

*Compulsory dining increases quality because the caterers know exactly what the uptake’s going to be… []… So, it just means that they can plan much more effectively to what they’re delivering….Actually the driver is purely and simply about nutrition. From my perspective is I can’t control what the children eat before school and after school but when we have such a high percentage of the students on free school meals, at least I know that they’re not just getting a load of rubbish or trying to save up that money in any way, shape or form.*

Non-intervention school staff

#### Self-efficacy

The final construct in Sekhon et al’s model of acceptability [27] is *self-efficacy*, participants’ capacity to participate in the intervention. UFSM was perceived to be more accessible than the national FSM scheme, both because many families may struggle financially but miss the threshold of eligibility for FSM, and difficulties applying for the scheme.

*It’s absolutely brilliant and even for parents who are working ‘cause I’ve been a working parent and struggled to pay. You know it’s not easy, there’s so much on bills and you know I’ve struggled to pay so I know it helps everybody.*

Y7 parent School 1

*One thing I’ve noticed in general is that there’s certain benefits that my son’s entitled that I’ve had to apply for. I know I’m a relatively well-educated person. English is my first language and it’s not straight forward. I remember when I was going through the application process that if I had problems with literacy or English was not my first language, it would be really hard and I think there’s probably a correlation between literacy and the need for free school meals so I could see that being a challenge for somebody.*

Y8 Parent School 2.

### Enablers and barriers to the effective implementation of UFSM in secondary schools

#### Barriers

The implementation of UFSM in this pilot began just as the COVID-19 pandemic was building momentum in the UK. The pilot began in January 2020, and schools were closed as a mitigation measure two months later. Repeated closures, student absences, and Year group ‘bubbling’ all prevented the implementation proceeding as intended. Staff were unable to fully understand how UFSM was being experienced, and amend the implementation as a result.

*You know it is a shame that the pandemic kind of hit when it did because we didn’t see the consistent build-up of take-up, of enjoyment. You know there’s a big chunk missing in terms of our own understanding of how it all went. So really consistent period of check-in with the pupils I think would have been really key.*

School 1 Staff

The perceived quality of the food on offer, and the choice of menu options, was a barrier to take up for some children and parents. This was not a consequence of the introduction of UFSM, and more apparent in School 2.

*We’ve had a few concerns* [prior to UFSM] * around you know chicken that tastes fishy. …[]… it’s the off cuts of chicken that are then smushed together to create almost a chicken breast style piece….By increasing the amount we spent, which was only able to come through the local authority agreeing to that, because obviously they’re covering the universal free school meals so we wanted to up the quality as part of this process and that was the agreement we could engage in.*

School 2 Staff

Most parents were happy with the quality of food provided under UFSM despite some initial reservations that the pilot may have caused the cost-per-meal paid to caterers to decrease and quality of provision to decline. However, parents of children with special educational needs (School 2) were more likely to report concerns about the quality of food on offer, and the choices available, often related to their children’s sensory needs or reduced ability to make ‘sensible’ choices or communicate their unhappiness with the food. Students interviewed who were not participating in UFSM stated the choice of the food on offer as the reason; many preferred home-cooked food or had particular likes and dislikes that were better catered for by continuing to bring a packed lunch.

Student perceptions of school lunches collated in the peer diaries were broadly positive about the quality of food, but less so on the quantity. Most responses indicated that the menu options available, and enjoyment of food were ‘ok or good’ in both schools. Photos returned to the research team by student researchers indicate that few students chose the ‘healthy’ menu options available, such as side vegetables and salads. Comments on the returned dairies indicate that having a wide range of options is important to students.

*I say good because the lunch people give you an option on salad which is healthy and you don’t always have to have a hot lunch, you can have a sandwich instead.*

Year 9 Student School 1

The data returned by student co-researchers during lunchtime observations included perceptions of food options, queueing times and portion size using a simple fixed-option response scale. We report this data in Table 6 below. The diary is an unvalidated tool, and the small sample of respondents who completed it makes the data unsuitable for quantitative analysis but is presented here in descriptive form only. It does provide, both in its development with students (who determined the questions to be included) and in the responses, some indication of satisfaction levels with aspects of the school lunch that mattered most to students. In School 1 many diary entries (see Table 6)

indicated satisfaction with the portion size, but eight (of thirty-seven responses) suggested it was too small. Portion size was a greater concern for students in school 2 where nine of twenty responses indicated dissatisfaction with the amount of food provided. Some responses also indicate that students do not think that the school lunch was big enough to provide energy for the rest of the school day. In school 1 some students (11 of 37 responses) were buying additional snack items bought in school during break or lunchtime. This may have reintroduced some inequity in access to food as not all students may be able to afford to purchase snacks. Dissatisfaction with the amount of food provided was not a result of the introduction of UFSM; nevertheless it was a potential barrier to participation (preferring packed lunches) or in other cases, encouraged students to buy snacks.

The student observations also revealed that while both schools provided ‘healthier’ menu options such as salads and side portions of vegetables, these were not popular amongst the students.

**Table 6:** Student observation results

		School 1 (37 responses)	School 2 (20 responses
Queuing time	*Ok*	23	10
	*Fast*	4	5
	*Too long*	10	5
Menu Options	*Ok*	22	12
	*Good*	11	5
	*Poor*	3	2
Bought items at lunch	*No*	28	n/a
	*Yes*	8	
Enjoyment of lunch	*Good*	13	7
	*Ok*	18	8
	*Poor*	4	2
Portion Size	*Too small*	8	9
	*About right*	26	9
	*Too large*	-	1
Enough energy for rest of day	*No*	8	6
	*Not sure*	13	6
	*Yes*	11	7
Snacks from home	*No*	30	n/a
	*Yes*	4	
Breaktime food purchase	*No*	23	n/a
	*Yes*	11	

#### Enablers

COVID-19 mitigation measures may have *enabled* the smooth introduction of UFSM by changing how students accessed the canteen at lunchtime. Efforts to keep students within Year Group bubbles meant that lunchtimes were staggered in both schools, reducing the number of students queuing for food and sitting in the canteen at any one time. This meant queuing times and demand for seats was reduced despite the increased proportion of students accessing school meals. However, students also noted that those accessing lunch at later sittings were more likely to face reduced choice as some menu options had already run out.

Communication to parents about UFSM at the launch of the pilot was important and required a lot of staff effort in both schools. Having members of the senior leadership team driving this process was a key enabler.

*She (Principal) really wanted it to work. Like she will pick and choose what she gets very involved in herself, because she’s a busy woman and all that, but she really was on this one and just nagging people and chasing it and chasing it and chasing it.*

School 2 Staff

Age was both an enabler and barrier; staff perceived that uptake of UFSM school meals was immediate for students joining the school in Y7. When presented to new students and their families, UFSM was seen as ‘standard’ and did not require change. Staff perceived that for parents of older students who had been at the school for longer, there was some degree of inertia and resistance to change.

*Parents had just – I guess there’s a conscious decision there not to engage and our year seven’s coming through with the offer in place, apart from those with significant sensory needs are going straight onto school dinners.*

School 2 staff

Finally, the provision of funds for School 2 to improve its kitchen area made it easier for the school to implement UFSM.

### What are the cost implications of UFSM?

UFSM cost the local authority £93,773 for the two participating schools in the 2020/2021 financial year, excluding infrastructure and staff costs. Costs for the 2021–20,221 are not reported due to the significant changes in uptake due to COVID-19 school closures. The estimated cost of providing UFSM for a child not eligible for FSM would be £449 per school year, assuming 100% uptake and attendance. Staff largely thought that UFSM was a good use of financial resources, and believed that the fundamental determinant of continuing UFSM provision was funding. It was seen as a small cost, with a potentially large impact. The opportunity costs of UFSM included extending lunchtime into lesson time, and more staff being required to supervise the lunch break.

*I think it’s a start, definitely. I think there’s certainly more that can be done, but again it all revolves around money and the money that schools are given to do these things, but it’s an absolute brilliant initiative that should be continued.*

Staff School 1

#### Universal vs. targeted provision

Staff and parent respondents had varied views about whether the cost of providing universal free school meals was a reasonable use of public funds. For some respondents, the cost of UFSM could be considered as another resource necessary for students to learn, and was an effective means of support for all children and young people. The cost of UFSM was perceived to be minimal when compared to other national expenditure:

*What has been shown is that it’s not a priority, not that it’s not feasible….[]…But when you talk about the education budget, the NHS budget, social care budget, the amount we’re paying on warheads, its miniscule. It’s absolutely minuscule to do it for everyone, let alone just for the people who need it. So, from that perspective, this is a choice we need to make as a country and about whether we’re willing to do it.*

School 1 staff

Those with concerns about universality worried about the overall cost, and also that benefits would accrue to wealthier families who did not need them. However most respondents with these concerns also recognised that means-tested free school meal provision would result in a cohort of families who missed the threshold for eligibility, or did not apply, and were left struggling with food insecurity. One staff respondent suggested that if UFSM was implemented more widely, wealthier families could be given an option to decline the benefit as a means of saving costs to the taxpayer. Most were willing to countenance ‘wasting’ benefits on families with higher incomes.

*One of the concerns I had was, because it’s a sort of blanket scheme where everyone gets a free meal, it feels like some people will be benefiting from it, who don’t necessarily need to, us included. Which feels like not a great use of resources but overall if you have to balance that with kids who live with food insecurity, it’s probably worthwhile. If it guarantees that those kids get a good meal, it’s probably okay to ‘waste’ some money on people who don’t need it.*

Y8 Parent School 1

*If we reverted to FSM, there would be more students who fall through cracks and do not claim FSM. I think there is no way of making them fully accessible. Like there’s no way of making those administrative systems really accessible genuinely to absolutely everybody, so yeah there would definitely be kids not getting fed.*

School 1 staff

There were also school staff who perceived that the cost of UFSM would only be justifiable if there was a strong evidence base for the impact on long-term health and educational outcomes for children and young people. Some of these respondents suggested alternative approaches to addressing food insecurity amongst secondary school pupils, such as giving schools funds that they could use to target families most in need, including those just missing the threshold for FSM. This would in effect lower the eligibility threshold for FSM but implementation would vary across schools.

#### Breakfast or lunch?

Some staff respondents had a preference for free breakfasts rather than lunches. Many noted that schools were already providing free or subsidised breakfasts because this was another meal that those students living in food- insecure households were likely to miss or skip. Some suggested that government funding should be provided for breakfast provision, rather than expecting schools to find the money from existing funds. Providing a free meal earlier in the day may bring optimal benefit as more children were coming to school hungry and would find it difficult to wait until lunchtime to eat. There was also a perception that some parents would find it more acceptable to miss breakfast, but would still ensure their children had access to lunch.

*I think it’s breakfast that they really miss out on. Like I think a lot of kids their whole life is just too chaotic, so I think for a lot of kids like they would eat a lunch one way or another anyway, like they’re not that dysfunctional – their families are not that dysfunctional; but I think a lot of kid’s families dysfunctional enough that they don’t eat breakfast, ‘cause just like again, it’s not blame – like you’ve got super busy parents…*

School 1 staff

Other school staff preferred lunch provision as a means of addressing hunger and food insecurity both because more students would benefit (many may not arrive in school on time to access breakfast; lunchtimes had a more ‘captive audience’), and that the nutritional value of lunch provision was higher than breakfast.

*Definitely lunch. I think lunch is more important you know, it’s a bigger meal, you have more of a variety at lunch, it’s not just beans on toast. You have the meat, you have the vegetables, you have more of a range of food so I think lunch would be more important.*

School 2 staff.

## Discussion

This study examined feasibility, acceptability, implementation, and cost implications of the introduction of universal free school meals in two London secondary schools using qualitative data collected from school staff, students, and parents. The findings suggest that UFSM is both a feasible and acceptable intervention for this age group. Staff, students and parents in the intervention schools were supportive of UFSM, broadly because of the coherence of the intervention. Indeed, there was also support from those staff interviewed from non-intervention schools. Increased access to a healthy meal, reduced food insecurity and better nutrition were clearly understood as the goals of the intervention. This clarity is in stark contrast to a previous study of the implementation UFSM in Scotland, where school staff were less supportive of the policy because of its perceived lack of coherence [29], or it not being considered the best way of supporting families most in need [19]. UFSM was also perceived as an effective intervention, particularly in increasing uptake of school meals and reducing the financial burden on families. The study has also demonstrated the feasibility of UFSM, albeit on a small scale. Both schools successfully implemented the initiative.

The acceptability of UFSM amongst parents was increased by their perception that the intervention supported low-income families not previously eligible for FSM but still with living with food insecurity. This has been seen in other studies of UFSM [30]. Cohen *et al’s* systematic review of UFSM found evidence for an increased uptake of meals for both students eligible for FSM, and those not [14]. Improving uptake for those previously eligible for FSM suggests that UFSM may be successfully addressing concerns about stigma and challenges with awareness of and/or applying for the FSM. Increased uptake amongst those previously not eligible for FSM suggest that UFSM is also supporting those families living with food insecurity but above the threshold for FSM. Increasing overall uptake of school lunch may confer the potential benefits of school meals, including better nutritional value (compared to packed lunch) and potential social and educational impacts on all students, not just those who are living with food insecurity. Students and parents in the current study were keen on the equity of the policy, providing the same access to school meals for all families.

There were some burdens and barriers to implementation, including increased queuing times. This was mitigated in the current study by staggered lunch breaks introduced as a COVID-19 prevention measure, and it is also a common means of reducing queuing times and managing space in schools with restrictive dining areas where UFSM has been implemented for younger children [31]. Other burdens identified in this study include infrastructure and staffing changes. These were successfully addressed in the current pilot in small secondary schools but may be more challenging for larger schools. Evaluation of the implementation of UFSM for primary-aged children in Scotland revealed that infrastructure improvements and increased staffing of kitchen and dining halls was also necessary for successful implementation [19]. These set-up burdens and costs may be short-term; further research is required to determine if the long-term gains outweigh the costs. It is feasible that catering companies may benefit from the economies of scale as a result of UFSM. School staff perceive that in the longer term UFSM is easier and less burdensome to administer than the FSM scheme.

Some of the perceived benefits of UFSM, such as increased uptake, improved nutrition and social skill development may also be achieved through the compulsory dining model described by one of the respondents in this study. We do not have data on the acceptability of this model (which is not the norm in UK secondary schools), though it is unlikely to achieve the same perceived financial benefits for low income families, or lower the reported burden of managing students at lunchtime under the standard FSM model reported by staff in UFSM intervention schools.

Catering to the dietary needs and preferences of all students, as well as managing portion sizes, were the main challenge to the acceptability of UFSM; both parents and students raised concerns about the lunch offer, particular for those children with special educational needs. The perceived quality of the school lunch offer may not be a direct consequence of UFSM but would need to be addressed to optimise the beneficial impacts for students and families. Involving students in menu planning may increase the acceptability of the lunch offer, reduce food waste, and encourage the inclusion of healthier options including side vegetables and salads that students are more likely to choose. Observation data shows that students rarely chose the ‘healthy’ option; if the nutritional benefits of UFSM are to be maximised the intervention may need to be supplemented with other school-wide activities to promote healthy eating that includes staff, students and parents. This may include the provision of better guidance and policies for schools on improving the nutritional quality of school meals [32].

Some staff participants in the current study had a preference for the provision of universal free breakfasts rather than lunches, to enable hungry students to eat earlier in the school day. There were also respondents who thought that lunch provision was likely to reach a greater proportion of students, and provide a better nutritional gain than breakfast. This latter view is supported by the Cohen et al. review which found that the association between UFSM and improvements in diet quality and academic performance were strong when lunch was included, but more mixed when only universal free breakfasts were on offer [14].

There remain concerns about the benefits of universal provision, as opposed to other ways of supporting food insecure families. While most respondents were in favour of UFSM, there were a small proportion who were conflicted between the desire to reduce food insecurity, and limiting the costs to the taxpayer. These respondents did not want to ‘waste’ public money on wealthier students and families who were not food insecure. Estimating the cost of FSM as £2.50 per student/school day, the UK Child Poverty Action Group suggest that the cost of rolling out UFSM nationwide, in addition to the schemes that already exist across the four nations, would be £1.8bn per year [33]. Universal provision of welfare has declined in the UK, but devolution in Scotland and Wales has seen a return to the approach, particularly welfare provision in relation to children [33]. Morelli and Seaman used data derived from the 2002 British Household Panel Study (BHPS) to examine the potential impact of a proposal to extend UFSM to all children in Scotland through the unsuccessful Free School Meals Bill in 2002. Their analysis found that targeted FSM was failing to support the lowest-income families because many did not meet the eligibility threshold for support or were not in receipt of the qualifying benefits, despite being eligible. Furthermore, in their model inequality was reduced as universality increased, providing FSM meals to all families in the lowest nine income deciles; only provision of FSM to the top income decile increased inequality across households. They suggest that the concern about UFSM benefiting middle and high income families and increasing inequality may be overstated; while universal provision does benefit the middle classes, this is offset by the advantages of reduced administration costs, reduction in stigma, and improved perception of FSM (as more middle-income families use them)[34]. There is growing public support for UFSM in the UK. A nationally representative poll of 10, 069 UK adults in November 2020 (during the height of the pandemic) found that 51% agreed with the statement “*school meals should be free for all students so that poor students are not stigmatised*” [35], though it is unclear if respondents were asked specifically about older, secondary school-aged students.

This is the first study of the implementation of universal free school lunch provision in secondary schools in the UK. It benefits from the inclusion of stakeholders from both intervention and non-interventions schools, and in particular, from the inclusion of the perception of students, both in qualitative interviews and as co-researchers conducting observations of their lunchtimes. It is limited by the small-scale pilot involving only two relatively small secondary schools within a single London borough. The participant sample was further restricted by the impact of the COVID-19 pandemic, including school closures and the study team relying on school staff to recruit and select parent and student respondents; this may have had a gatekeeping effect, introducing some bias. The lunchtime observation diaries enabled students to participate as co-researchers and the capture of observations that were salient to students but are a previously untested method using an unvalidated tool. The data available on costs of the pilot was limited and excluded staffing and infrastructure costs. It would also have been useful to speak to a larger number of non-intervention school staff.

Wider adoption and implementation of UFSM in secondary schools may only be achieved if there is a stronger evidence base for the impact on long-term health and educational outcomes. This is supported by a study of the implementation of UFSM in Scotland which recommends that building the evidence base for the impact of UFSM on outcomes that are meaningful to education stakeholders is necessary for the long-term success of UFSM [28]. We would add that evidence of long-term impact on all health, psychosocial and education outcomes is liable to appeal to practice and policy stakeholders across a range of domains, as well as assuaging the concerns of some that benefits were ‘wasted’ on higher-income families. Measuring these long-term outcomes would also support a more detailed cost-effectiveness analysis.

## Conclusion

This process evaluation of universal free school meal provision in two UK secondary schools finds evidence of acceptability and feasibility amongst students, parents and school staff involved in the pilot as well as school staff in non-intervention schools. Perceived benefits of UFSM include increased access to a healthy meal, reduced food insecurity and better nutrition for students, as well as the social benefits derived from communal eating. UFSM is also perceived as successfully supporting families experiencing food insecurity but not eligible for means-tested free school meals. Barriers to implementation, including school infrastructure that limits the number of students who can eat at any one time, and demands on catering staff, also seen in other studies of UFSM, were largely overcome. Although not a consequence of introducing UFSM, parent and student concerns over menu choice and quality will need to be addressed if the intervention is to achieve optimal uptake and nutritional benefits for students. Future studies of UFSM in UK secondary schools should include a larger and more diverse sample of schools, and include long-term all health, education and psychosocial outcomes so that the impact and cost effectiveness of the approach can be determined and compared with other interventions.

## Electronic supplementary material

Below is the link to the electronic supplementary material.

Supplementary Material 1

## Data Availability

Anonymised qualitative transcripts used during the current study are available from the corresponding author on reasonable request.

## List of abbreviations

FSM: Free School Meals
NIHR: National Institute for Health Research
PHIRST: Public Health Interventions Research Studies Teams
UFSM: Universal Free School Meals
USDA: United States Department of Agriculture
