# Supplementary material for: A qualitative process evaluation of universal free school meal provision in two London secondary schools
Source: BMC Public Health. 2023 Feb 9;23:300. doi: 10.1186/s12889-023-15082-3 (PMC9910769; doi:10.1186/s12889-023-15082-3)
Supplement: Supplementary file 1 — Supplementary Material 1 [file 12889_2023_15082_MOESM1_ESM.pdf]

Co-researcher lunchtime diary

Your name:

Year:

Date of observation:

Day of the week:

Arrival time (in canteen):

Time spent queuing for food:

Total time spent in canteen:

Total time for lunch break:

Please use all of the tick boxes and only fill in extra details if you want to or have time.

1. **What was the queue like?**

(too long) ☐

(ok) ☐

(fast) ☐

Reasons for any delay

……

……

……

2. **What was on the menu today?**

……

……

……

3. What do you think about the menu options?

(Good)

(OK) ☐

(Poor) ☐

Why do you say this?

………

………

……..

4. Were there both hot and cold options?

(Yes) ☐

(No) ☐

5. Do you think there were enough options for people who have special diets e.g. halal, vegetarian, vegan, other preferences

(Yes) ☐

(No) ☐

Please say more:

………

………

……..

5a Did you bring a pack lunch in today?

(Yes) ☐

(No) ☐

Please say why:

……

……

……

6. What did you have to eat and drink at lunch?

Main meal…………………………………………………………………………………………….

Pudding……………………………………………………………………………………………….

Snacks…………………………………………………………………………………………………..

Drinks…………………………………………………………………………………………………

7. Did you pay for anything?

(Yes) ☐

(No) ☐

7a Please give details of what is currently available to buy

……

……

………..

8. Please tell us about the cost of anything you a paid for:

£ *(Cheap)* ☐

£ £ *(About right)* ☐

£ £ £ *(Too expensive)* ☐

8a. Were the costs easy for you to see?

👍 *(Yes)* ☐

👎 *(No)* ☐

9. Did you enjoy your food?

😋 *(Yum!)* ☐

😐 (OK) ☐

😩 *(No)* ☐

tell us why:

………

………

………

10. Overall, what rating would you give your food today? (1-5 stars)

☆ ☆ ☆ ☆ ☆

11. Did you eat all of it? (photo of lunch tray at start and end of meal)

(Yes) ☐

(No) ☐

Tell us why:

………

………

………

12. What did you think about the portion size (amount of food served)

(too small) ☐

(about right) ☐

(too much) ☐

13. Did you bring extra food in from home to eat at any time today e.g. snacks?

(Yes) ☐

(No) ☐

Details

………

………

………

14. Did you buy anything else from the canteen today?

At break time:

(*Yes*) ☐

(*No*) ☐

At lunch time:

(*Yes*) ☐

(*No*) ☐

Details

………

………

………

15. Do you think your lunch will give you enough energy to be able to concentrate and work for the rest of the afternoon?

(*Yes*) ☐

(*No*) ☐

(*Not sure*) ☐

16. Was there a good choice of drinks? 📷

Free drinks:

😔 *(Poor choice)* ☐

😐 *(Ok)* ☐

😋 *(Good choice)* ☐

Drinks you pay for:

😔 *(Poor choice)* ☐

😐 *(Ok)* ☐

😋 *(Good choice)* ☐

tell us more

………

………

………

17. Was there a good choice of condiments e.g. salt and pepper, sauces etc available? 📷

👍 *(Yes)* ☐

👎 *(No)* ☐

Details (e.g. what others would you like?).

……………………………………………………………………………………………………

……………………………………………………………………………………………………

……………………………………………………………………………………………………

18. Were there enough plates, cups and cutlery available?

(Yes) ☐

(No) ☐

Please say more

……

……

……

19. Were you able to sit where you wanted to eat?

(Yes) ☐

(No) ☐

Please say more (e.g. where would you like to be able to sit)

……

……

……

20. Were you able to sit with your friends?

(Yes) ☐

(No) ☐

Please say more

……

……

……

21. Would you have been able to take your food outside if you wanted to?

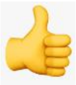 *(Yes)* ☐

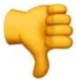 *(No)* ☐

22. Think about how long you spent in the canteen. Was this enough time?

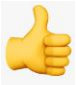 *(Yes)* ☐

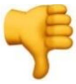 *(No)* ☐

Please say more

……

……

……

23. What did you do for the rest of lunchtime after you finished eating?

……

……

……

24. What were the good things about lunch today?

………

………

………

25.What things that could have been better?

………

………

………

Please tell us anything else we haven’t thought of and you’d like to say:

………

………

………

………

………

………

………

………

………

………

………

………

………

………

……

……

……

……

……

……

……

……

……

……

……

……

……

……

……

……

……

……

……

……

……

……

Thank you so much for doing this today!!!

Please hand your form to ….
